# Sodium butyrate ameliorates diabetic retinopathy in mice via the regulation of gut microbiota and related short-chain fatty acids

**DOI:** 10.1186/s12967-023-04259-4

**Published:** 2023-07-07

**Authors:** Yinhua Huang, Zhijie Wang, Bo Ye, Jacey Hongjie MA, Shangli Ji, Wang Sheng, Suna Ye, Yiwen Ou, Yanfang Peng, Xu Yang, Jiansu Chen, Shibo Tang

**Affiliations:** 1 Aier School of Ophthalmology, Central South University, Changsha, China; 2 Aier Eye Institute, Changsha, China; 3 State Key Laboratory of Ophthalmology, Optometry and Visual Science, Eye Hospital, Wenzhou Medical University, Wenzhou, China; 4 National Clinical Research Center for Ocular Diseases, Eye Hospital, Wenzhou Medical University, Wenzhou, China; 5 Department of Ophthalmology, Nanchang Aier Eye Hospital, Nanchang, China; 6 Department of Ophthalmology, Changsha Xiangjiang Aier Eye Hospital, Changsha, China; 7 Key Laboratory for Regenerative Medicine, Ministry of Education, Jinan University, Guangzhou, China; 8 Changsha Aier Eye Hospital, Aier Eye Hospital Group, Hunan, China

**Keywords:** Diabetic retinopathy, Gut microbiota, Short-chain fatty acids, Sodium butyrate, 4-Methylvaleric acid, Caproic acid

## Abstract

**Background:**

Diabetic retinopathy (DR) development is associated with disturbances in the gut microbiota and related metabolites. Butyric acid is one of the short-chain fatty acids (SCFAs), which has been found to possess a potential antidiabetic effect. However, whether butyrate has a role in DR remains elusive. This study aimed to investigate the effect and mechanism of sodium butyrate supplementation on DR.

**Methods:**

C57BL/6J mice were divided into three groups: Control group, diabetic group, and diabetic with butyrate supplementation group. Type 1 diabetic mouse model was induced by streptozotocin. Sodium butyrate was administered by gavage to the experimental group daily for 12 weeks. Optic coherence tomography, hematoxylin–eosin, and immunostaining of whole-mount retina were used to value the changes in retinal structure. Electroretinography was performed to assess the retinal visual function. The tight junction proteins in intestinal tissue were evaluated using immunohistochemistry. 16S rRNA sequencing and LC–MS/MS were performed to determine the alteration and correlation of the gut microbiota and systemic SCFAs.

**Results:**

Butyrate decreased blood glucose, food, and water consumption. Meanwhile, it alleviated retinal thinning and activated microglial cells but improved electroretinography visual function. Additionally, butyrate effectively enhanced the expression of ZO-1 and Occludin proteins in the small intestine. Crucially, only butyric acid, 4-methylvaleric acid, and caproic acid were significantly decreased in the plasma of diabetic mice and improved after butyrate supplementation. The deeper correlation analysis revealed nine genera strongly positively or negatively correlated with the above three SCFAs. Of note, all three positively correlated genera, including *norank_f_Muribaculaceae*, *Ileibacterium*, and *Dubosiella*, were significantly decreased in the diabetic mice with or without butyrate treatment. Interestingly, among the six negatively correlated genera, *Escherichia-Shigella* and *Enterococcus* were increased, while *Lactobacillus*, *Bifidobacterium*, *Lachnospiraceae_NK4A136_group*, and *unclassified_f_Lachnospiraceae* were decreased after butyrate supplementation.

**Conclusion:**

Together, these findings demonstrate the microbiota regulating and diabetic therapeutic effects of butyrate, which can be used as a potential food supplement alternative to DR medicine.

**Supplementary Information:**

The online version contains supplementary material available at 10.1186/s12967-023-04259-4.

## Introduction

Diabetic retinopathy (DR) is one of the most common and serious complications of diabetes. A meta-analysis of diabetic individuals revealed that the overall prevalence was 24.6% for any kind of DR, 6.96% for proliferative DR, 6.81% for diabetic macular edema, and 10.2% for vision-threatening DR [1]. The prevalence of any DR endpoint was higher in people with type 1 diabetes (T1D) than in people with type 2 diabetes [1]. Vision loss from DR has been a major and leading cause of blindness in adults, but to date, there is still no effective treatment available for it. Recently, it was reported that the gut microbiota plays a role in the occurrence and development of diabetes. Our previous population study found that the development of DR is closely related to gut microbial dysbiosis [2]. With advances in research, the gut microbiota has increasingly become a new target for the prevention and treatment of DR [3]. In diabetes patients, the abundance of probiotics in the intestinal tract has been shown to decrease, while the level of opportunistic pathogens increased significantly. In addition, potentially pathogenic microbes and microbial products were also identified in the retina of the T1D mouse model [4]. An experimental animal study revealed that dietary interventions could prevent the development of DR in mice by altering the composition of the gut microbiome [5]. However, there are currently few studies using gut microbiota or its related metabolites as a treatment for DR.

Short-chain fatty acids (SCFAs) are the major metabolites produced by the gut microbiota fermentation of dietary fiber. Microbial SCFA production has significant effects on improving gut integrity and modulating host metabolic health, such as activating G protein-coupled receptors, improving fasting hyperglycemia, and delaying the development of diabetes [6, 7]. A previous study showed that diabetic mice have significantly decreased SCFA levels compared to normal mice [8]. Of note, the relevance of circulating rather than fecal SCFAs in regulating insulin sensitivity, lipolysis, and GLP-1 concentrations in humans has been reported [9]. Furthermore, a recent study demonstrated that a reduction in plasma SCFA was associated with significant changes in intestinal morphology and microbiome composition, and these gut changes may contribute to metabolic disorders and accelerate the onset and development of T1D in mice [10]. Studies conducted in humans with long-standing T1D have shown that metabolite-based dietary supplementation increases SCFA acetate, propionate, and butyrate concentrations in stool and plasma, leading to a shift in the composition and function of the gut microbiota and better glycemic control [11].

Animal experiments confirmed that plasma SCFA levels were decreased in T1D mice, with the most pronounced decline in butyrate [10]. Butyric acid is one of the SCFAs and is essential for maintaining host health by providing energy for the intestinal epithelium, increasing the expression of tight junction proteins in colon epithelia, exhibiting anti-inflammatory effects, regulating the immune system, and affecting different metabolic pathways throughout the body [12, 13]. Sodium butyrate has been proven to reduce blood glucose by reducing pancreatic β-cell damage, insulin resistance, and gluconeogenesis [14]. In addition, due to butyrate stabilizing the tight junctions of the gastrointestinal epithelium, lower butyrate concentrations may further promote intestinal barrier instability [15]. Given that butyrate has anti-inflammatory, anti-glycemic, and reinforced barrier activity, it may have potential therapeutic activity against DR.

This study aimed to explore the ameliorative effect of sodium butyrate on DR and gut microbiota. Our study first analyzed the effect of butyrate on systemic lesions in diabetic mice by measuring body weight, blood glucose, food, and water consumption. In addition, we observed morphological and functional changes in the diabetic retina and small intestine. Furthermore, the changes in systemic SCFA concentration and gut microbiota were explored using correlation analysis. Our study provides a valuable new approach that not only alleviates retinal thinning and microglial cell activation but also improves the intestinal barrier and gut microbiota dysbiosis. Sodium butyrate may be a potential therapy for the treatment of DR.

## Materials and methods

### Experimental animals and design

Five- to six-week-old male C57BL/6J mice (SPF grade) were purchased from Hunan SJA Laboratory Animal Co., Ltd. (Changsha, China) and housed in ventilated microisolator cages under a 12 h:12 h light:dark cycle, humidity at 50 ± 15%, and temperature at 22 ± 2 °C. The small intestinal tissues were assigned to become diabetic or remain nondiabetic controls. Intraperitoneal injection of streptozotocin (STZ) in sodium citrate buffer (pH 4.3) was used to induce diabetes in mice at the age of 6 weeks (50 mg/kg STZ of body weight for five consecutive days). The control group was intraperitoneally injected with an equal volume of sodium citrate buffer. The onset of diabetes was defined as any blood glucose level higher than 16.7 mmol/L in blood collected from each mouse’s tail [16]. Mice were randomly divided into three groups (n = 5–7 per group): nondiabetic control (control group), diabetic control (T1D group), and diabetic with butyrate supplementation (T1D + NaB group). The OCT and ERG measurements being conducted on parallel groups of animals (n = 5 each). The three groups were fed a normal chow diet. Sodium butyrate was given by gavage administration (500 mg/kg body weight) to T1D + NaB group once a day continuously for 12 weeks. Body weight and blood glucose levels were recorded every 4 weeks. Food and water consumption were recorded each week.

All animal experimental procedures in this study adhered to the Association for Research in Vision and Ophthalmology Statement for the Use of Animals in Ophthalmic and Vision Research and were approved by the Animal Ethics Board of Central South University (CSU-2023-0014).

### Optic coherence tomography (OCT)

To monitor retinal morphological changes in vivo, OCT scanning was conducted using the micron IV system (Phoenix Research Labs, USA) after butyrate treatment for 12 weeks. Mice were anesthetized by intraperitoneal injection of pentobarbital sodium (40 mg/kg), and the eyes were dilated with *tropicamide phenylephrine* eyedrops (Santen). OCT imaging was performed using the high-definition circular scan mode. Total retinal thickness was defined as the distance from the distal edge of the retinal pigment epithelium (RPE) layer to the proximal edge of the nerve fiber layer (NFL). Further segmented retinal layers included the inner layer (from the NFL to the proximal edge of the inner nuclear layer (INL), the middle layer (from the INL to the inner and outer segments (IS/OS) of the photoreceptors), and the outer layer (from the end of IS/OS to the RPE). Each retinal thickness was measured using Image-pro Plus software.

### Hematoxylin–eosin (HE) and immunohistochemical (IHC) staining

The right eyeball of the mice was removed after butyrate supplementation for 12 weeks and fixed with FAS eyeball fixation (Servicebio, G1109) for 24 h at room temperature, followed by subsequent dehydration using graded ethanol and embedding in paraffin. A 4 μm thick horizontal section through the optic nerve head was achieved using a microtome (RM2016, Leica, Heidelberg, Germany). The tissues were stained with standard H&E and examined for morphometry. The retinal structure stratifications of the total retina, inner layer, middle layer, and outer layer were the same as OCT, and the thickness was measured using Image-pro Plus software.

The small intestinal tissues were removed and fixed with 4% paraformaldehyde at 4 °C overnight, then dehydrated sequentially in 20 and 30% sucrose solutions for 24 h. Subsequently, the tissues were embedded in optimal cutting temperature compound and cut into 10 µm sections. IHC staining was detected by streptavidin peroxidase (*SP*) *kit* (SP0041, Solarbio, Beijing, China). The sections were rinsed twice with phosphate-buffered saline to remove optimal cutting temperature compound and then treated with 3% H_2_O_2_ for 10 min at room temperature to quench endogenous peroxidase activity. After being blocked with normal goat serum for 20 min at room temperature. The sections were incubated overnight at 4 °C with antibodies against ZO-1 (1:250 dilutions, 33-9100, Invitrogen) and Occludin (1:250 dilutions, 13409-1-AP, Proteintech), and then incubated with biotinylated goat anti-mouse or anti-rabbit secondary antibodies at 37 °C for 30 min. All antibodies were diluted in the diluent provided by the *SP kit*. Finally, diaminobenzidine was used to visualize staining, and a hematoxylin (Biosharp Co., Ltd, China) counterstain was applied to the tissue slides. The histological sections were evaluated under a light microscope at 400× magnification.

### Immunostaining of whole-mount retinas

The left eyeball of the mice was removed for whole-mount retina immunofluorescence. Briefly, enucleated eyes were fixed in 4% paraformaldehyde for 30 min on ice. After dissection of the retina, the tissues were incubated in Iba1 primary antibody (1:400, Novus, NB100-1028), which was diluted in 3% bovine serum albumin + 0.5% Triton X-100 + 5% goat serum overnight at 4 °C. Then, retinal tissues were incubated in the secondary goat anti-rabbit Alexa Fluor 488 antibody (A-11034, Invitrogen, USA). After flat mounting, retinal immunofluorescence was imaged using an LSM800 confocal microscope (Zeiss, Germany), and the whole mount retina structure was analyzed using ImageJ software.

### Electroretinography (ERG)

ERG measurements were performed after butyrate supplementation for 12 weeks. The mice were stimulated by Ganzfeld Q450 SC (ROLAND) after overnight dark adaptation. Mice were anesthetized with pentobarbital sodium (40 mg/kg), the pupils were dilated with *tropicamide phenylephrine* eyedrops (Santen), and mice were placed on a temperature-regulated heating pad throughout each recording session. The recording ring electrode was placed in the center of the corneal surface, the grounding electrode was connected to the tail root, and the negative electrode was placed on the back of the neck. The lower light intensities (0.0095, 0.095, and 0.95 cds/m^2^) were averaged from three light flashes interspaced over 30 s. The next flash stimulus (0.95 cds/m^2^) was for the OPS, and after bright adaptation for 1.5 min, flashes with stimuli (3.00 cds/m^2^) were used. The amplitude of the a-wave was measured after flash onset from the first lowest peak to the prestimulus baseline, while the amplitude of the b-wave was measured between the first lowest peak and the first highest peak. Oscillatory potentials (OPs) were automatically filtered out by ERG software. The latencies and amplitudes of OP1-3 were measured separately.

### Detection of SCFAs concentrations

A total of eight SCFAs, including acetic acid, propionic acid, isobutyric acid, butyric acid, isovaleric acid, valeric acid, 4-methylvaleric acid, and caproic acid, in plasma samples were analyzed using liquid chromatography‒mass spectrometry/mass spectrometry (LC–MS/MS) at Majorbio Bio-Pharm Technology Co. Ltd. (Shanghai, China). Briefly, plasma samples (50 μl) were mixed with acetonitrile to final concentration of 50% acetonitrile. SCFA were derived for 30 min at 40 °C with 20 μl of 200 mM 3-nitrophenylhydrazine and 20 μl 120 mM N-(3-dimethylaminoprony)-N′-ethylcarbodiimede hydrochloride. For LC–MS/MS detection, the mix was diluted to 750 μl with 50% acetonitrile aqueous solution. The LC–MS/MS analysis of sample was conducted on a ExionLC AD system coupled with a QTRAP 6500+ mass spectrometer (AB Sciex, USA). The mass spectrometer was operated in negative mode using electrospray ionization source operating. Quality control samples were inserted in samples during the instrumental analysis. LC–MS/MS raw data were collected and processed using AB Sciex quantitative software to drawn a Linear regression standard curve. The mass spectrum peak area of the sample analyte was substituted into the linear equation to calculate the concentration.

### Fecal microbiota analysis

Fecal samples were collected into sterilized collection tubes and flash-frozen in liquid nitrogen, then stored at − 80 °C until further analysis. Total microbial DNA was extracted from the fresh feces of mice using the E. Z.N.A.^®^ Soil DNA Kit (Omega Bio-Tek, Inc.). The microbial 16S rDNA was amplified using the forward primer 5′-ACTCCTACGGGAGGCAGCAG-3′ and the reverse primer 5′-GGACTACHVGGGTWTCTAAT-3′ with the Illumina MiSeq sequencing system based on the Ribosomal Database Project with a 2 × 250 bp paired-end method after the library was quantified, mixed and quality checked. All the results were based on sequenced reads and operational taxonomic units (OTUs) with a 97% similarity cutoff. The sequences were analyzed using the QIIME34 (Quantitative Insights Into Microbial Ecology) software package. In-house Perl scripts were used to analyze α-diversity and β-diversity. Phylogenetic Investigation of Communities by Reconstruction of Unobserved States analysis was performed to predict the differential fecal microbiota based on 16S rRNA sequences.

### Statistical analysis

Data were analyzed with Microsoft Excel 2016 and GraphPad Prism 8.0 software (GraphPad Software, Inc., San Diego, CA, USA). Normally distributed data were expressed as the mean ± SEM, the Shapiro–Wilk test was used to assess the normality of data distribution, and significance was identified using one-way ANOVA. Non-normal data were tested using the Kruskal–Wallis H test and FDR was applied to correct for the multiple comparisons. Correlation analysis was performed using Spearman correlations. *p* < 0.05 was considered statistically significant.

## Results

### Effect of butyrate on physiological indices of diabetic mice

**Fig. 1:**
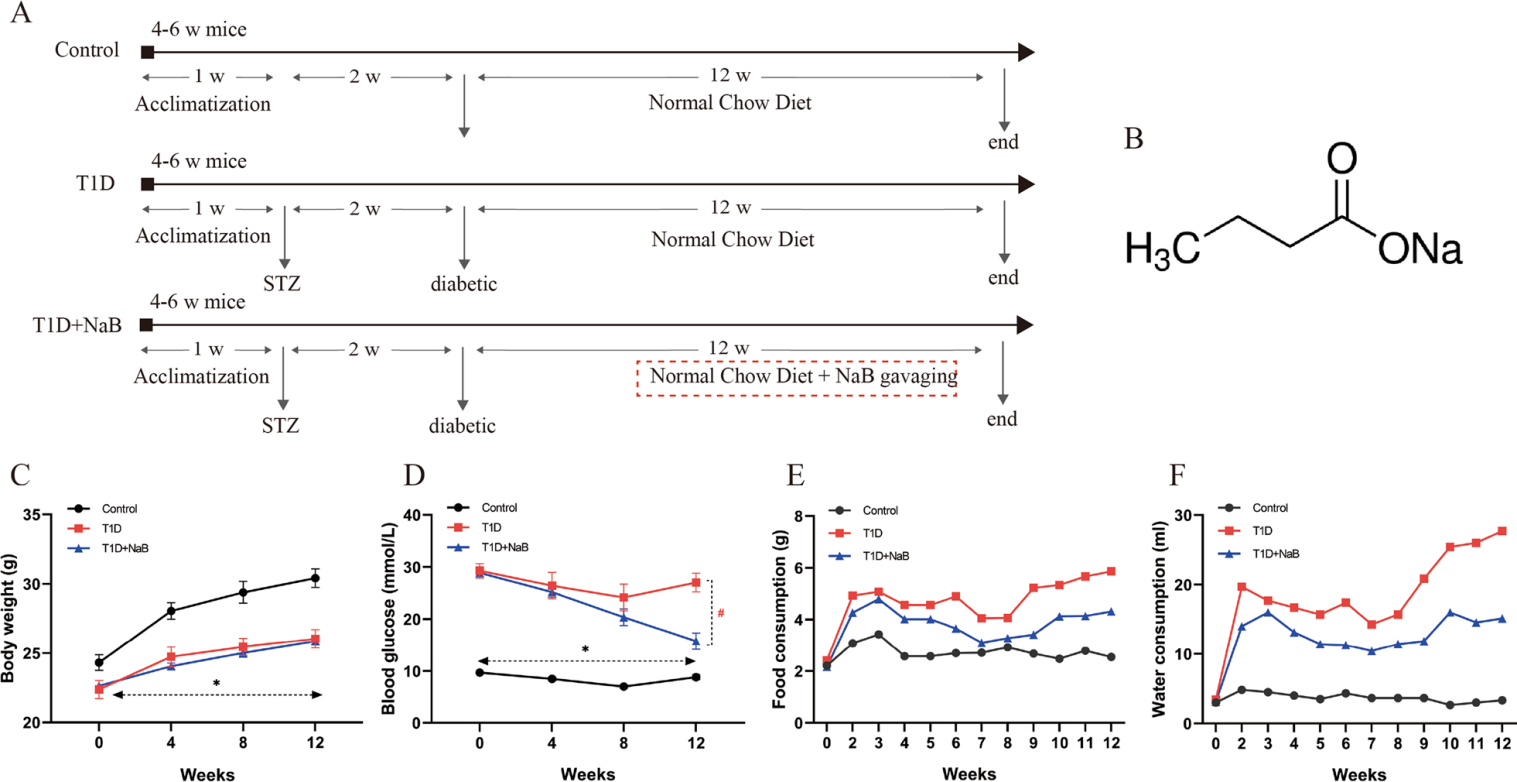
Effect of butyrate on physiological indices of diabetic mice. **A** Schematic diagram of animal treatment. **B** Molecular structure of compound sodium butyrate (NaB). **C** Body weight, **D** blood glucose, **E** food consumption, and **F** water consumption levels of all mice were monitored for the intervening period of the study. Data represent the mean ± SEM, n = 5–7 per group, *p < 0.05 vs. control, ^#^*p* < 0.05 vs. T1D group

In this study, the diabetic mouse model was induced by STZ. To understand the effect of butyrate on the body, we measured several physiological indices. Body weight and blood glucose levels were measured every 4 weeks, while food and water intake were measured weekly over the 12-week sodium butyrate treatment period. The whole experimental process is schematically depicted in Fig. 1A. The formula name of sodium butyrate is C_4_H_7_NaO_2,_ and its molecular structure is shown in Fig. 1B. We found that the two diabetic groups with STZ injection showed a significant decrease in body weight gain compared with the control, while there was no significant difference between the diabetic groups with and without butyrate treatment (Fig. 1C). Blood glucose levels in control mice were in the normal range of 7–10 mmol/L, while levels in diabetic mice were in the range of 15–30 mmol/L. Diabetic mice in the butyrate supplementation group had lower blood glucose levels than the diabetic control group, and a significant effect on blood glucose levels was observed at 12 weeks (Fig. 1D). The daily food and water consumption levels of diabetic mice were higher at each time point than those of control mice, and both were decreased after butyrate supplementation (Fig. 1E, F). Butyrate reduced blood glucose levels, food, and water consumption but not body weight in diabetic mice.

### Butyrate ameliorated retinal thinning in diabetic mice

**Fig. 2:**
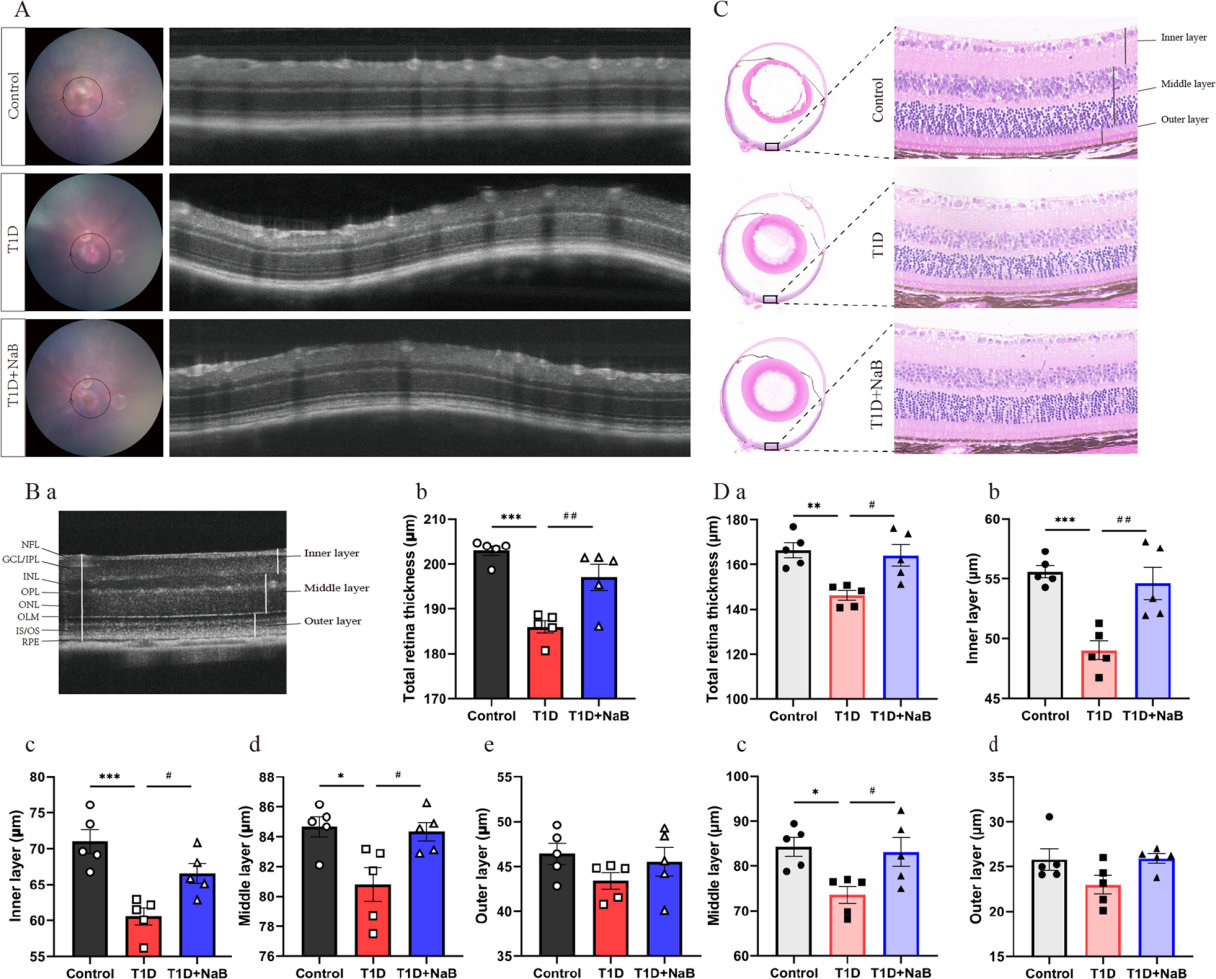
Optical coherence tomography (OCT) examination and hematoxylin and eosin (HE) staining revealed retinal alterations in diabetic mice. **A** Representative OCT image. **Ba** The definition of the retinal layers in OCT images. **Bb**–**Be** The thickness of the total retina, inner layer, and middle retinal layer decreased in the T1D group compared with the control group but increased in the T1D + NaB group. The thickness of the outer retinal layers was not different among the three groups. **C** Representative retinal morphology images of HE staining. **Da**–**Dd** The thickness of the total retina, inner layer, and middle retinal layer decreased in the T1D group compared with the control group but increased in the T1D + NaB group. The thickness of the outer retinal layer was not different among the three groups. The data are presented as the mean ± SEM (n = 5). *p < 0.05, **p < 0.01, ***p < 0.001 compared with the control group. ^#^*p* < 0.05, ^##^*p* < 0.01, ^###^*p* < 0.001 compared with the T1D group. Scale bar: 100 μm for **A**, Scale bar: 20 μm for **C**

To determine the effects of butyrate on ocular histological morphology, we used OCT and HE staining to observe the retinal morphology and measure the thickness of the different layers in the retina. A schematic of the retinal layers is shown in Fig. 2Ba. Representative structural images of the OCT examinations for the three experimental groups are shown in Fig. 2A. The thickness of the total retina was decreased in the T1D group compared with the control group but increased in the T1D + NaB group (Fig. 2Bb). Further analysis of segmented retinal layers in the T1D group and T1D + NaB group showed that structural alterations occurred mainly in the inner layer and middle retinal layer. Butyrate treatment significantly ameliorated the T1D mouse reduction in retinal thickness in the inner layer and middle layer (Fig. 2Bc, d). The thickness of the outer retinal layers was not different among the three groups (Fig. 2Be). We further analyzed retinal tissue sections with HE staining. Consistently, the representative retinal morphology images of HE staining showed that the thicknesses of the total retina, inner layer, and middle layer in T1D mice were decreased compared to those in control mice, and these thicknesses increased after butyrate supplementation (Fig. 2C, Dc). Similarly, there was no difference in the thickness of the outer layer of the retina in the three groups (Fig. 2Dd). Thus, histological analyses via OCT and HE staining demonstrated that butyrate ameliorated retinal thinning in diabetic mice, notably improving the total retina, inner layer, and middle retinal layers.

### Butyrate inhibited microglial activation in diabetic mice retinas

**Fig. 3:**
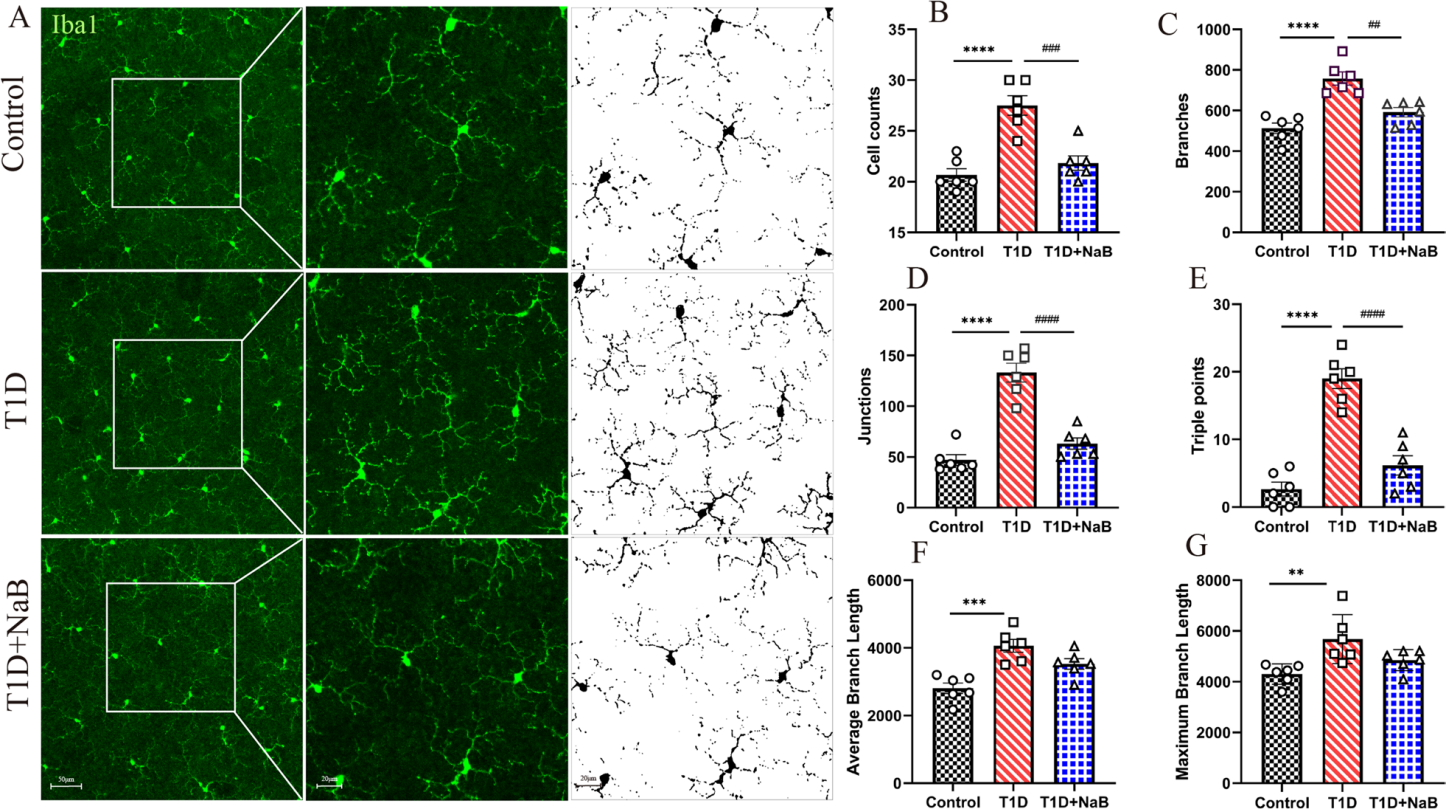
Butyrate inhibited microglial activation in diabetic mouse retinas. **A** Morphological changes in microglia in the three groups. **B**–**G** Results of comparison of cell counts, branches, junctions, triple points, average branch length, and maximum branch length quantities. The data are presented as the mean ± SEM (n = 6). *p < 0.05, **p < 0.01, compared with the control group. ^##^*P* < 0.01 compared with the T1D group. Scale bars: 50 μm and 20 μm

A previous study reported that microglial cells in the retina were identified by Iba1, which was upregulated during the inflammatory activation of microglia. Microglial activation was assessed based on cell morphology, with resting microglia exhibiting small bodies and activated microglia presenting mean large cell bodies [17]. To explore the effect of butyrate on retinal microglia, whole retina staining of Iba1 was performed after butyrate supplementation for 12 weeks and is shown in Fig. 3A. We found that whole retina staining showed microglial activation, including increasing the cell number, branches, junctions, and triple points in the T1D group compared with the control group. However, microglial activation decreased in the T1D + NaB group (Fig. 3B–E). The average branch length and maximum branch length in the T1D group were significantly increased compared with those in the control group. However, the changes in these two indices did not reach statistical significance but displayed a decreasing trend in the T1D + NaB group compared with the T1D group (Fig. 3F, G). The results suggested that butyrate inhibits microglial activation by attenuating altered cell morphology and returning them to normal microglial cells in the retinas of diabetic mice.

### Butyrate improved ERG visual function in diabetic mice

**Fig. 4:**
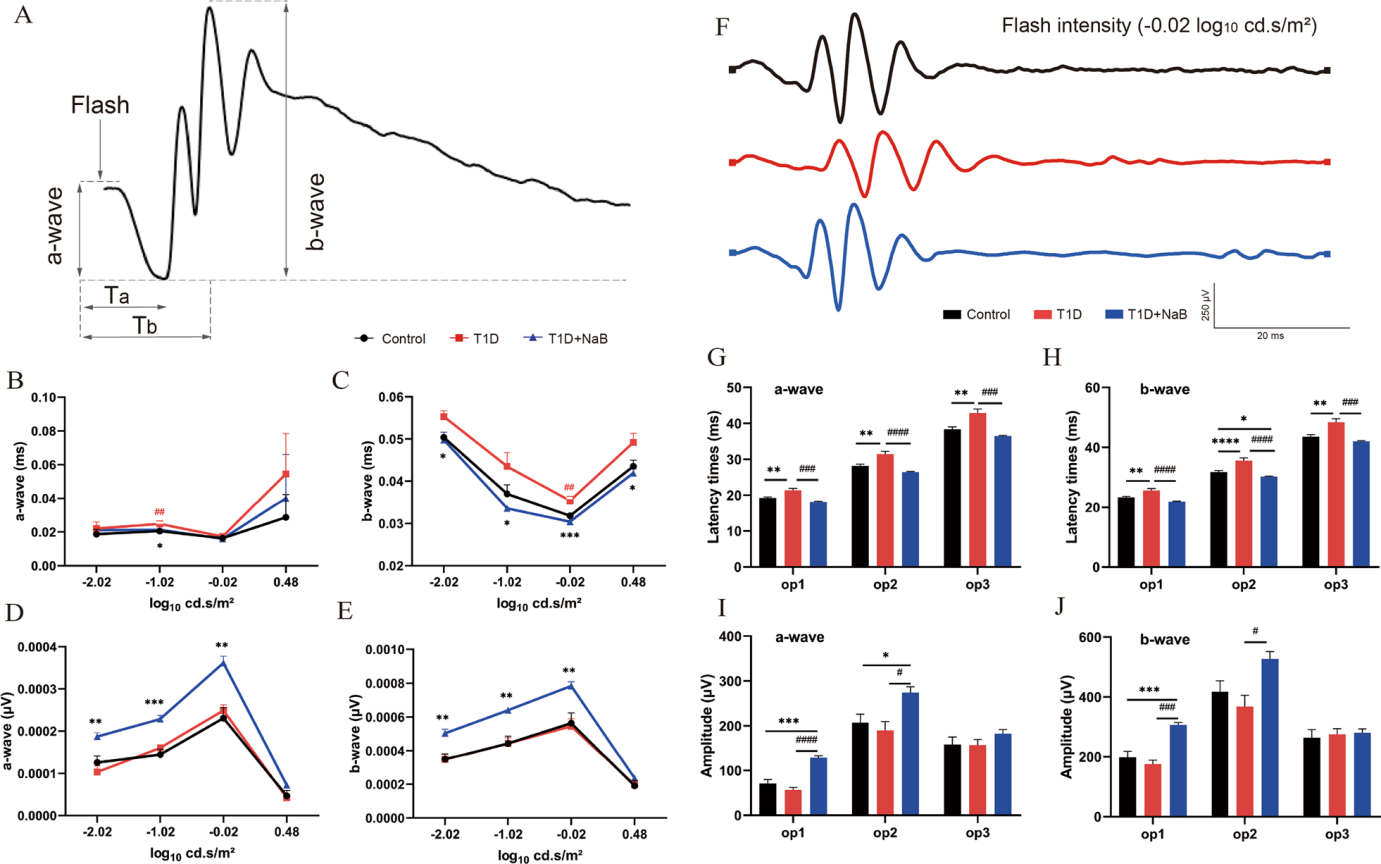
Butyrate improved ERG visual function in diabetic mice. **A** Double-beaded arrows reaching toward the dashed lines represent a- and b-waves. **B**, **C** The implicit times of the a-wave and b-wave were increased in the T1D group compared with the control group, and this change was reversed in the T1D + NaB group. **D**, **E** The changes in a-wave and b-wave amplitudes were not obvious between the T1D and control groups. **F** Representative results of OPs are shown for the control (black line), T1D (red line), and T1D + NaB (blue line) groups. **G**, **H** The OP latency times of the T1D group were significantly increased compared with those of the control group, while these changes were significantly reversed in the T1D + NaB group. **I**, **J** There were no differences in OP amplitude between the T1D group and the control group, while OP1 and OP2 significantly increased in the T1D + NaB group compared with the T1D group. The data are expressed as the mean ± SEM (n = 5). *p < 0.05, **p < 0.01, ***p < 0.001, ****p < 0.0001. ERG, Electroretinography

To evaluate the effect of butyrate on retinal function in diabetic mice, ERG was applied after butyrate supplementation for 12 weeks. Representative ERG and OP results are shown for the control (black line), T1D (red line), and T1D + NaB (blue line) groups (Fig. 4F, Additional file 1: Fig. S1). Double-beaded arrows reaching toward the dashed lines represent the a-wave and b-wave (Fig. 4A). The implicit times of the a-wave (photoreceptors) and b-wave (bipolar cells and M $\ddot{u}$ ller glial cells) were increased in the T1D group compared with the control group. However, this change could be reversed by administering butyrate under different stimulus intensities (Fig. 4B, C). The changes in a-wave and b-wave amplitudes were not obvious between the T1D and control groups. Intriguingly, the amplitudes of the a-wave and b-wave were significantly higher in the T1D + NaB group (Fig. 4D, E). The OP (amacrine cell) latency times of the T1D group were significantly increased compared with those of the control group, while these changes were significantly reversed in the T1D + NaB group (Fig. 4G, H). There were no differences in OP amplitude between the T1D group and the control group, while OP1 and OP2 significantly increased in the T1D + NaB group compared with the T1D group (Fig. 4I, J). These findings demonstrated that butyrate supplementation could alleviate diabetic-related visual function damage by reducing implicit times and increasing amplitudes of a-waves and b-waves, as well as reducing latency time and increasing amplitude of OPs.

### Butyrate enhanced the expression of tight junction proteins in the small intestine

**Fig. 5:**
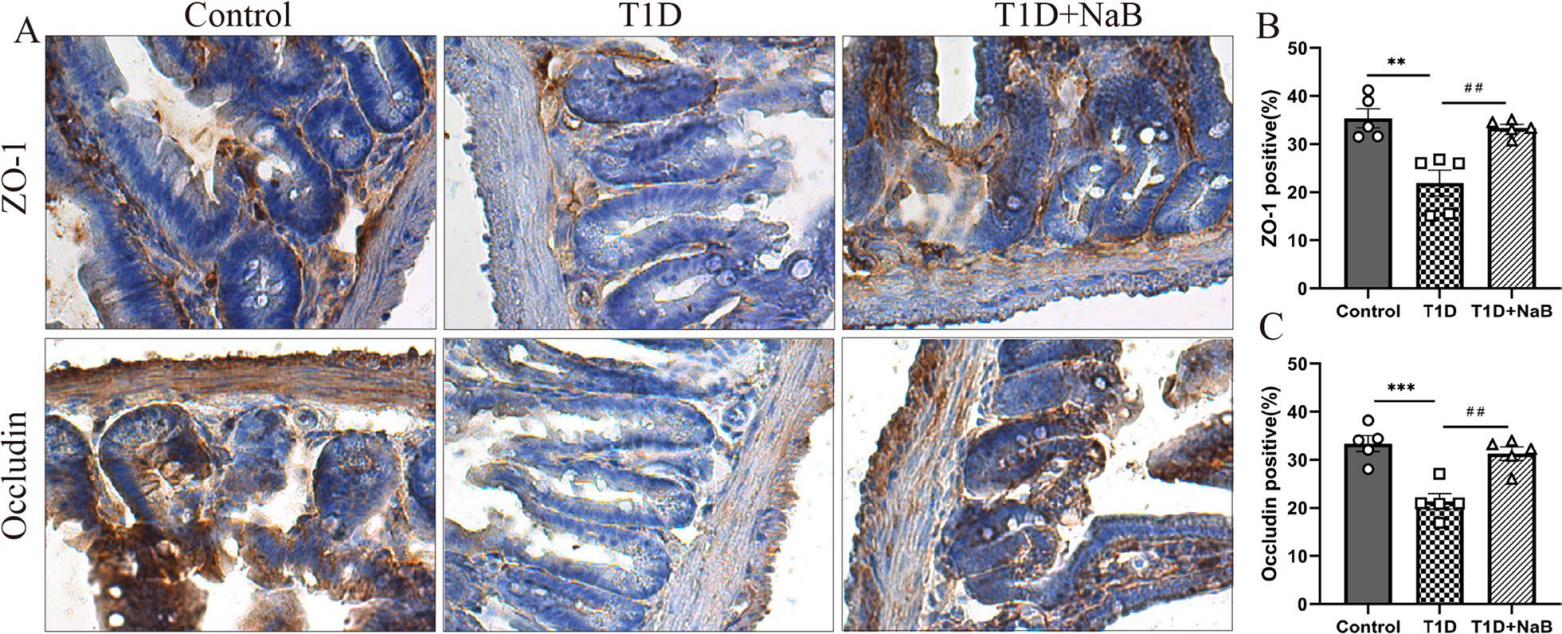
Butyrate enhanced the expression of tight junction proteins in the small intestine. **A** Representative immunohistochemical staining results of ZO-1 and Occludin proteins in the small intestine. **B** The expression of ZO-1 and Occludin proteins significantly decreased in the small intestine of the T1D group compared with the control group, while it increased in the T1D + NaB group. The data are presented as the mean ± SEM (n = 5). **p < 0.01, ***p < 0.001, compared with the control group. ^##^*P* < 0.01 compared with the T1D group

To determine the effects of butyrate on the tight junction proteins in the small intestine, IHC staining was performed to detect the expression of tight junction proteins, including ZO-1 and Occludin. As shown in the results, both ZO-1 and Occludin proteins were located at the apex of the villous enterocytes and expressed abundantly in the control group, while these two proteins significantly decreased in the small intestine of diabetic mice (Fig. 5A, B). Meanwhile, a marked elevation in ZO-1 and Occludin protein expression was observed in the small intestine of the T1D + NaB group (Fig. 5B). These results suggested that butyrate may improve intestinal barrier function by enhancing the expression of ZO-1 and Occludin proteins.

### Butyrate increased systemic SCFA levels in diabetic mice

**Fig. 6:**
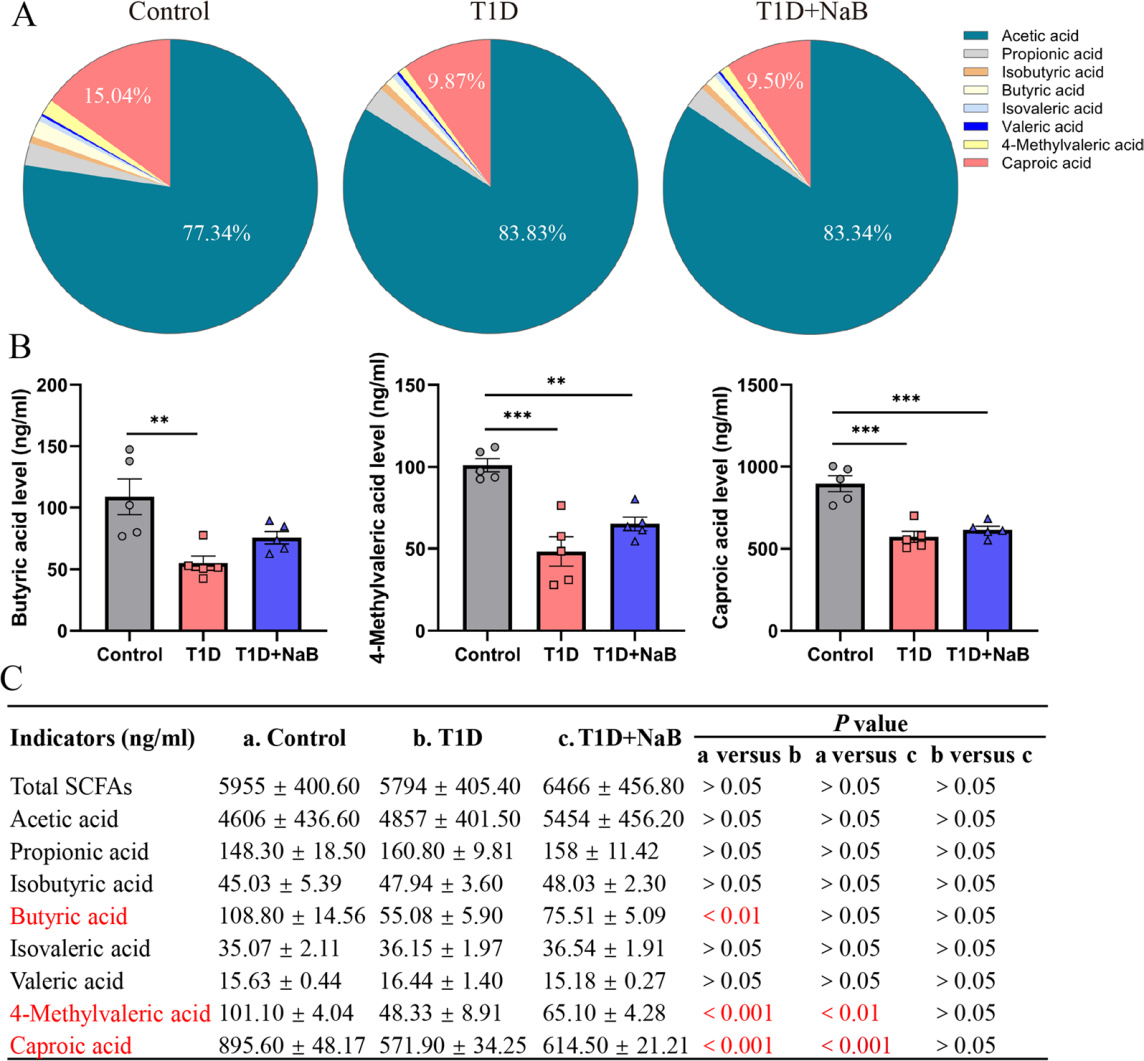
Butyrate increased systemic SCFA levels in diabetic mice. **A** The pie chart shows the relative proportions of SCFAs in the T1D, T1D + NaB, and control groups. **B** Butyric acid, 4-methylvaleric acid, and caproic acid levels in plasma significantly decreased in the T1D group compared with the control group. **C** The concentrations of total and individual SCFAs were calculated. Data are presented as the mean ± SEM. SCFAs, Short-chain fatty acids. **p < 0.01, ***p < 0.001, n = 5

To evaluate the effect of butyrate supplementation on systemic SCFA levels, we measured the concentration of SCFAs in plasma at the endpoint time of the experiment. A total of eight SCFAs, acetic acid, propionic acid, isobutyric acid, butyric acid, isovaleric acid, valeric acid, 4-methylvaleric acid, and caproic acid, were examined for each sample using LC–MS/MS. According to the analysis of plasma SCFA profiles, both acetic acid and caproic acid mainly accounted for more than 90% of the total SCFA concentration in each group (Fig. 6A). Of all eight SCFAs detected, only three reached statistical significance. Specifically, the levels of butyric acid, 4-methylvaleric acid, and caproic acid in plasma were significantly decreased in the T1D group, and these three SCFA concentration declines were mitigated by butyrate supplementation but did not reach statistical significance (Fig. 6B). On the other hand, the levels of other SCFAs, including acetic acid, propionic acid, isobutyric acid, isovaleric acid, and valeric acid, as well as total SCFA levels, were not significantly different among the three groups. The concentrations of total and individual SCFAs were calculated as shown in Fig. 6C. Collectively, these data supported that diabetes influenced the proportions of systemic SCFAs, and butyrate supplementation in diabetic mice could attenuate the reduction in systematic butyric acid, 4-methylvaleric acid, and caproic acid levels in plasma.

### Sequencing data validation and diversity analysis of gut microbiota

**Fig. 7:**
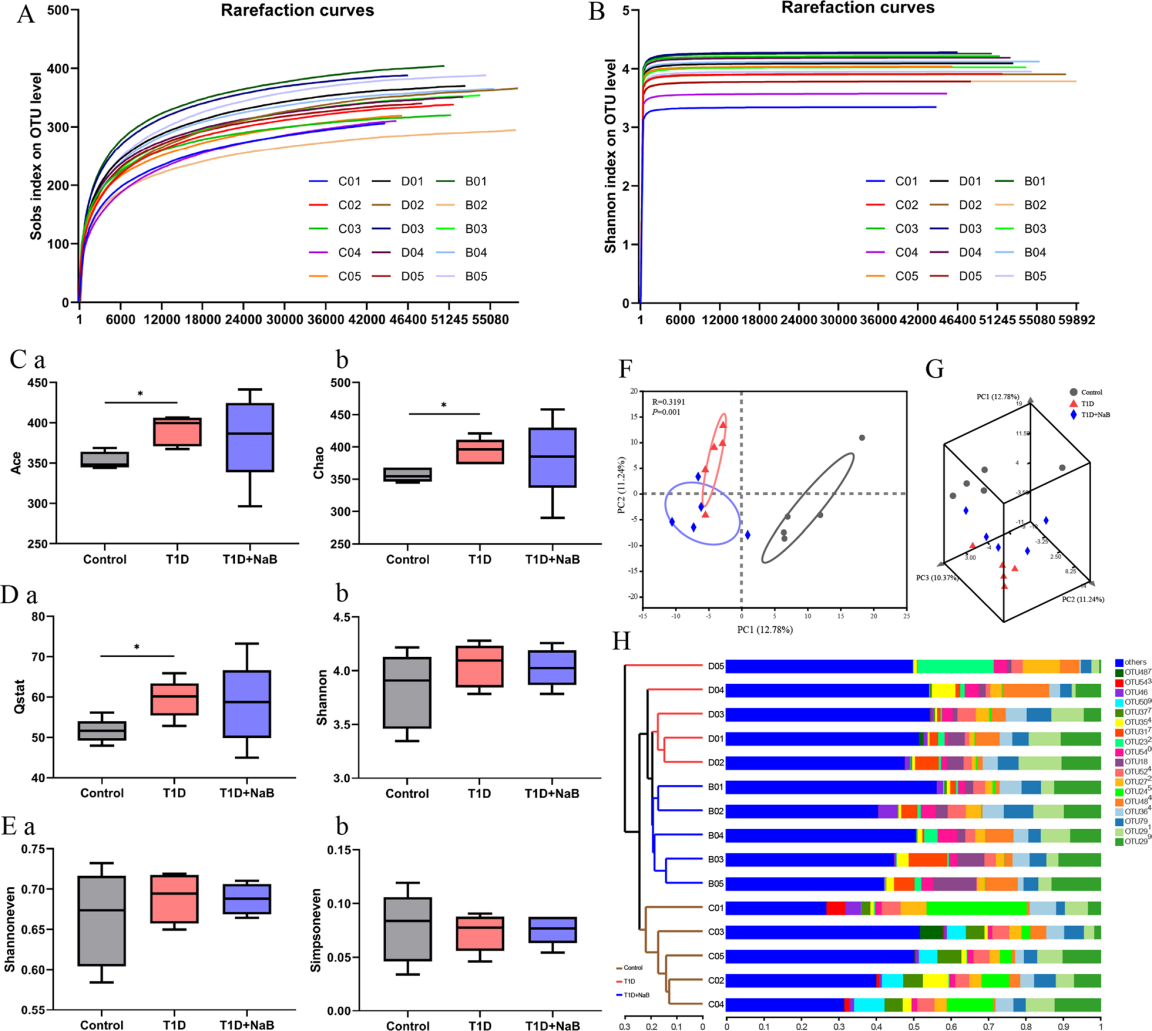
Sequencing data validation and diversity analysis of gut microbiota. Sobs rarefaction curve (**A**) and Shannon rarefaction curve (**B**) were drawn by the OTU number sampled. Indices of richness (**Ca**,**b**), diversity (**Da**,**b**), and evenness (**Ea**,**b**) estimators of the T1D, T1D + NaB, and control groups were used for the α-diversity analysis. 2D (**F**) and 3D (**G**) Principal component analysis plots comparing sample distribution in the three groups. **H** Hierarchical clustering at the OTU level. Data are presented as the mean ± SEM. *p < 0.05, n = 5

In the present study, the V3–V4 variable region sequences of gut microbiota in mice were classed according to their similarities. By random sampling of these sequences, the Sobs rarefaction curve was constructed based on the number of species sampled and the corresponding represented OTU (Fig. 7A). The Shannon rarefaction curve was presented by the microbial diversity index of each sample at different sequencing depths (Fig. 7B). Both the Sobs and Shannon rarefaction curves tended to be flat, indicating that the amount of sequencing data was large enough to reflect the vast majority of microbial information in all experimental samples. The analysis of α-diversity reflected the abundance and diversity of the microbial community in the T1D, T1D + NaB, and control groups. Based on the OTU level, the indices of Ace and Chao (Fig. 7Ca, b), Qstat and Shannon (Fig. 7Da, b), and Shannoneven and Simpsoneven (Fig. 7Ea, b) separately represented the species richness, diversity, and evenness. A box plot was plotted to support the findings of the data indicating higher α-diversity in the T1D group. Of these six metrics, the Ace, Chao, and Qstat indices were significantly increased, while the diversity index (Shannon) and evenness indices (Shannonenen and Simpsoneven) revealed slight changes in the diversity of microbes in the T1D group compared with the control group. However, compared with the T1D group, the T1D + NaB group showed partial restoration of gut microbial dysbiosis.

Principal component analysis (PCA), based on the evolutionary distance between species, is usually used to reflect the differences in microbial community composition among biological samples. The more similar the sample composition, the closer the distance that was reflected in PCA. As shown in the 2D and 3D PCA analyses (Fig. 7F, G), the gut bacterial community structures of the T1D and T1D + NaB groups were separated from that of the control group at the OTU level (R = 0.3191, *p* = 0.001). Hierarchical clustering also revealed that a statistically significant distinction was present between the microbiota of the three groups (Fig. 7H). It was suggested that the diversity and composition of the gut microbiota in diabetic mice are significantly altered, and additional butyrate treatment could reverse the gut bacterial alterations.

### Bacteria contributed to gut microbiota variety in the three groups

**Fig. 8:**
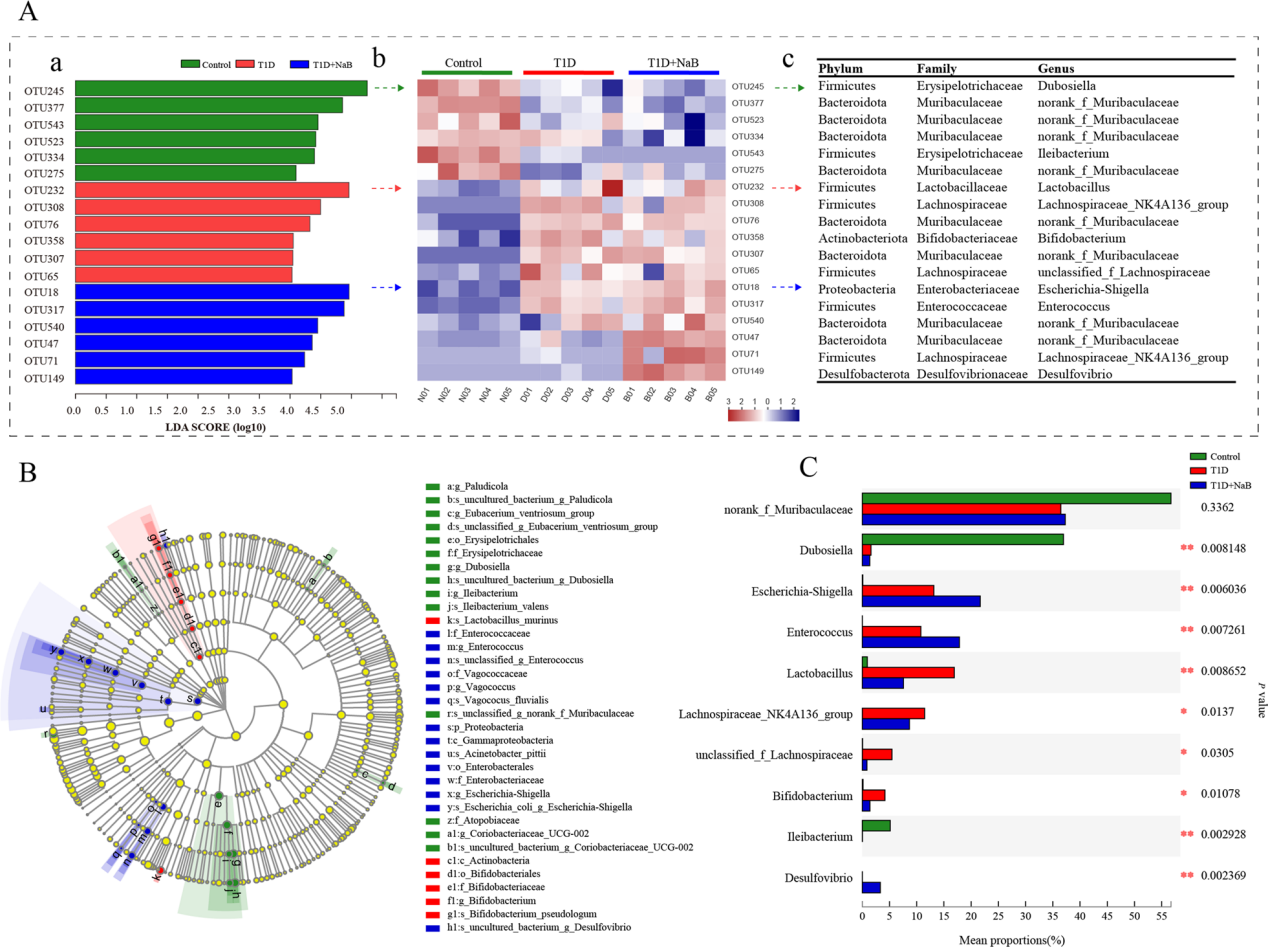
Identification of bacteria contributing to gut microbiota variety in the three groups using LDA and LEfSe. **Aa**–**c** LDA of bacteria at the OTU level (LDA > 3.5, heatmap (log10) corresponding to their taxonomy at the phylum, family, and genus levels, respectively. **B** Cladogram of bacteria at the OTU level (LDA > 3.5). **C** Kruskal‒Wallis H test bar plot at the genus level. Data are presented as the mean ± SEM. *p < 0.05. n = 5. LDA, linear discriminant analysis; LEfSe, linear discriminant analysis effect size

To understand the detailed changes in the gut microbiota, we identified the representative gut microbiota in the three experimental groups through linear discriminant analysis effect size (LEfSe) analysis. In total, 18 OTUs were identified with a log LDA score set above 3.5. The alterations in the bacteria at the OTU level were analyzed with LDA (Fig. 8Aa) and a heatmap (Fig. 8Ab). The OTUs corresponding to their taxonomy at the phylum, family, and genus levels are shown in Fig. 8Ac. Six OTUs (two belonging to Firmicutes and four belonging to the Bacteroidetes phylum) were highlighted in the control group. At the same time, 6 OTUs (three belonging to Firmicutes, two belonging to Bacteroidetes, and one belonging to the Actinobacteria phylum) and 6 OTUs (two belonging to Firmicutes, two belonging to Bacteroidetes, one belonging to Proteobacteria, and one belonging to the Desulfobacterota phylum) were highlighted in the T1D and T1D + NaB groups, respectively. The representative and high abundance gut microbiota in the T1D + NaB group were *Escherichia-Shigella*, *Enterococcus, norank_f_Muribaculaceae, Lachnospiraceae_NK4A136_group*, and *Desulfovibrio* at the genus level (Fig. 8Ac).

Based on the cladogram plot of LEfSe analysis from the phylum to species levels, the most significant gut microbiota clade in the T1D group was the Actinobacteria class to *Bifidobacterium pseudologum* species clade. In contrast, the dominant gut microbiota clades in the T1D + NaB group included the Proteobacteria phylum to *Escherichia_coli_g_-Shigella* species clade, Enterococcaceae family to *unclassified_g_Enterococus* species clade, and Vagococcaceae family to *Vagococus_fluvialis* species clade. These different dominant clades in the two groups showed the effect of butyrate treatment on remodeling the gut microbiome of diabetic mice (Fig. 8B). For all 18 OTUs screened in Fig. 8A, the Kruskal‒Wallis H test was used at the genus level and identified 10 genera in total. All but *norank_f_Muribaculaceae* showed that differences were statistically significant. The relative abundances of *norank_Muribaculaceae*, *Dubosiella*, and *Ileibacterium* were high in the control group, *Lactobacillus*, *Lachnospiraceae_NK4A136_group*, *unclassified_f_Lachnospiraceae*, and *Bifidobacterium* were significantly increased in the T1D group, and *Escherichia-Shigella*, *Enterococcus*, and *Desulfovibrio* were significantly increased in the T1D + NaB group (Fig. 8C).

### Correlation analysis between dominant gut microbiota and SCFA

**Fig. 9:**
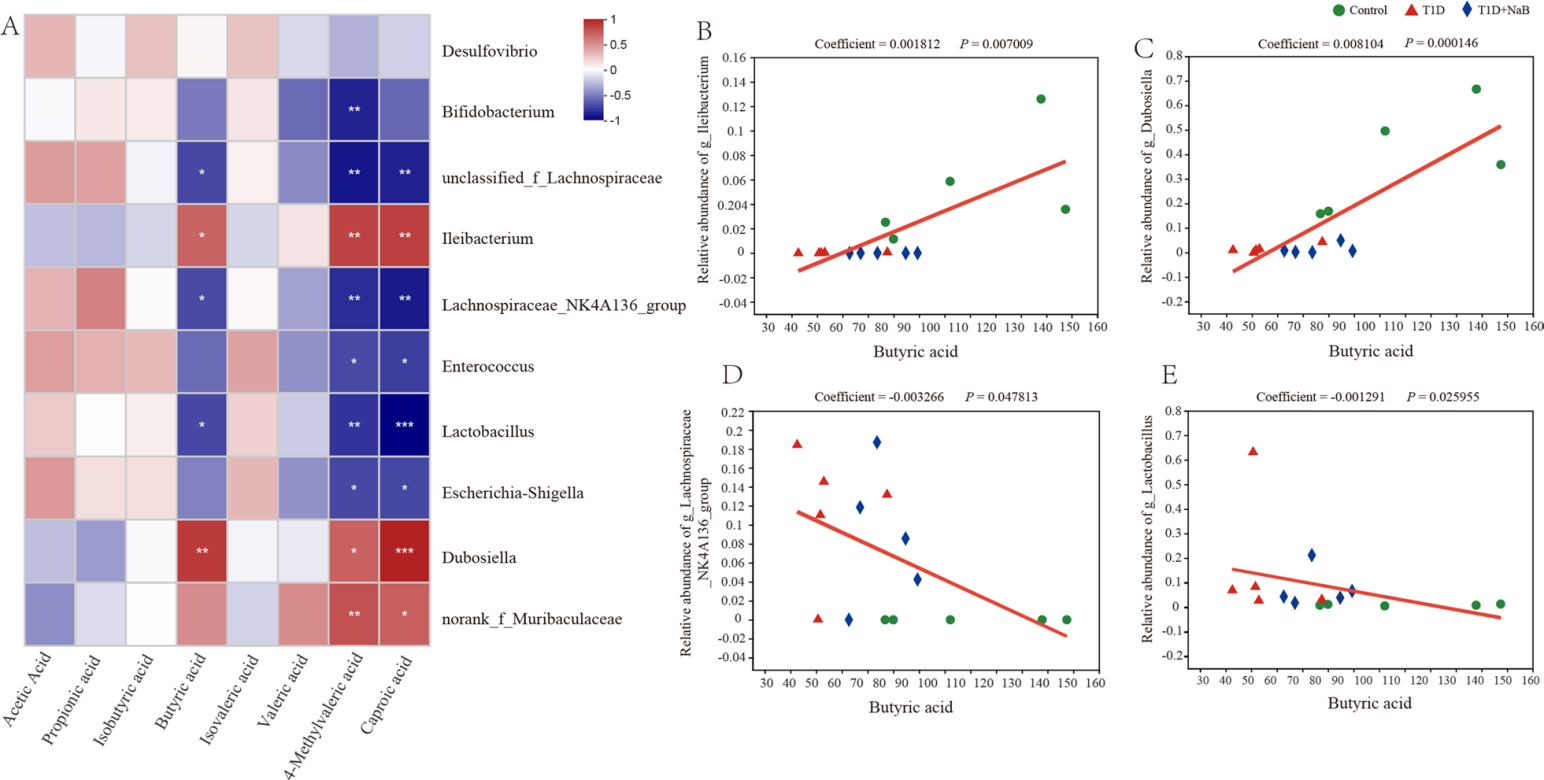
Correlation analysis between dominant gut microbiota and SCFA. **A** The correlation between the dominant changed gut microbiota at the genus level and SCFAs is displayed in the heatmap. The red‒white-blue grid corresponds to the value of (− 1)-to-1. Red color, positive correlation; blue color, negative correlation. Correlations were calculated in MaAsLin analysis: **B** *Ileibacterium* (coefficient = 0.001812, *p* = 0.007009), **C** *Dubosiella* (coefficient = 0.008104, *p* = 0.000146), **D** *Lachnospiraceae_NK4A136_group* (coefficient = − 0.003266, *p* = 0.047813) and **E** *Lactobacillus* (coefficient = − 0.001291, *p* = 0.025955). The color and shape of points represent individuals in each group. *p < 0.05, **p < 0.01, ***p < 0.001. n = 5. MaAslin, Multivariate Association with Linear Models

To assess whether there was a potential association between the altered gut microbiota and host metabolism, we analyzed the correlation between the relative abundance of the dominantly changed gut microbes at the genus level and SCFA in the plasma of mice using Spearman correlation analysis. The result was displayed in the form of a heatmap, which indicated that *Ileibacterium* and *Dubosiella* were significantly positively correlated with the levels of butyric acid, 4-methylvaleric acid, and caproic acid. *Unclassified_f_Lachnospiraceae*, *Lachnospiraceae_NK4A136_group*, and *Lactobacillus* genera were all significantly negatively correlated with the levels of butyric acid, 4-methylvaleric acid, and caproic acid (Fig. 9A). To further confirm the association between the abundance of bacterial genera and plasma butyric acid, 4-methylvaleric acid, and caproic acid levels in the three groups, we conducted multivariate association with linear model (MaAslin) analysis. For five bacterial taxa that showed a significant correlation with butyric acid shown in the heatmap, four of them also presented a significant correlation (all *p* < 0.05) using MaAslin analysis. Among them, the relative abundance of the *Ileibacterium* and *Dubosiella* genera in the control group was the highest, while their relative abundance in the T1D group was the lowest; both showed a positive correlation with butyric acid (Fig. 9B, C). In contrast, the other two genera showed opposite results; the relative abundances of *Lachnospiraceae_NK4A136_group* and *Lactobacillus* were negatively correlated with butyric acid, both of which were highest in the T1D group and lowest in the control group (Fig. 9D, E). In addition, the relative abundance of six and seven genera showed a significant association with 4-methylvaleric acid and caproic acid, respectively (Additional file 2: Fig. S2, Additional file 3: Fig. S3). Both genera that were enriched in the control group showed a positive correlation with 4-methylvaleric and caproic acid (Additional file 2: Fig. S2A, B, Additional file 3: Fig. S3A, B), while four and five genera enriched in the T1D group showed a negative correlation with 4-methylvaleric and caproic acid, respectively (Additional file 2: Fig. S2C–F, Additional file 3: Fig. S3C–G).

The above results suggested that the changes in gut microbes in diabetic mice mainly affected the butyric acid, 4-methylvaleric acid, and caproic acid levels of the host. In diabetic mice, the microbiota (*Ileibacterium*, *Dubosiella*, and *norank_f_Muribaculaceae genera*) partially positively correlated with the three SCFA production decreased, while the microbiota (*Bifidobacterium, unclassified_f_Lachnospiraceae*, *Lachnospiraceae_NK4A136_group*, *Enterococcus*, *Lactobacillus*, and *Escherichia-Shigella genera*) partially negatively correlated with the three SCFA production increased, and such changes could be mitigated by butyrate supplementation.

## Discussion

Diabetic retinopathy is one of the most common blinding complications of diabetes, and its pathogenesis is still not well understood. In the present study, sodium butyrate supplementation was examined as a strategy for DR therapy. Our results demonstrated that butyrate can effectively alleviate the histopathological changes of DR in vivo. Butyrate was able to reduce blood glucose and microglial activation, as well as improve the retinal structure and visual function in diabetic mice. In addition, small intestinal barrier function was enhanced, and gut microbiota dysbiosis was ameliorated after butyrate supplementation. Importantly, butyrate improved systematic SCFA levels, including butyric acid, 4-methylvaleric acid, and caproic acid level abnormalities, by reshaping the related gut microbiota composition. In summary, our findings suggest that butyrate might have a therapeutic effect to slow down the development of DR.

In this study, we used STZ injection to establish a T1D mouse model. Many of the hallmark pathophysiological changes, such as decreased body weight and increased blood glucose, food and water consumption, were observed in diabetic mice, as expected. All these manifestations are consistent with previous studies [18, 19]. Blood glucose decreased and reached a significant difference after butyrate supplementation for 12 weeks, while butyrate treatment failed to change body weight. In addition, butyrate slightly reduced the food and water consumption compared to the diabetic control. This is because butyrate oral administration could reduce appetite by suppressing the activity of orexigenic neurons in the hypothalamus and decreasing neuronal activity in the brainstem, thus leading to decreased food intake [20]. Intriguingly, Reports have shown that the changes of gut microbiota composition and weight loss can be caused by a calorie restriction (CR) diet. The results regarding CR-induced alterations in the relative abundances of main phyla bacteria including decreased Bacteroidetes and increased Firmicutes [21]. However, in the present study, all mice were fed with ad libitum chow diet and water. More importantly, the food and water consumption difference between two groups was not statistically significant. Hence, the gut microbiota composition and weight were little affected by reduced food and water intake.

Moreover, we found that retinal structure and function were impaired in T1D mice. The pathological results included thinning of the retina, activated microglial cells, and abnormal ERG in the retina. These findings were consistent with previous pathology results of T1D [22, 23]. Encouragingly, the pathological changes described above were improved after butyrate supplementation. Notably, there were several placebo-controlled clinical trials of butyrate treatment in T1D did not find an impact. De Groot et al. [24] found that oral butyrate supplementation for 1 month did not result in any significant changes in adaptive or innate immunity, or in any of the other outcome variables in humans with longstanding T1D. Similarly, Tougaard et al. [25] reported that 12 weeks of butyrate supplementation did not reduce intestinal inflammation in persons with T1D, albuminuria, and intestinal inflammation. Our data in mouse models of T1D seem to contrast previous studies showing no effect of butyrate in human T1D. This may be explained by the variations in disease severity. The animal models in our study are in the early stage of diabetes, while human trials are largely in populations with longstanding disease with severe diabetic complications. Another possible explanation is the differences in administration mode and dose, the timing and duration of the intervention related to the stage of the disease process. Additionally, the differences in physiology, pathology, and intestinal microbiology between species may result in different therapeutic effects. Thus, exploring these issues may help us understand the role of butyrate in early T1D and direct further research.

Accumulating literature has shown that STZ induction leads to gut bacterial dysbiosis and intestinal barrier interruption in diabetic mice. For example, a recent study revealed that a notable separation of β-diversity and differences at the phylum, family, and genus taxonomic levels in STZ-induced T1D compared with control mice [19]. The Ace, Chao, and Qstat indices were used to calculate the species richness and diversity of the microbiota, whereas PCA and hierarchical clustering trees reflected the magnitude of population differences between groups. Combined with the two aspects, our results showed that the gut microbiota of diabetic mice was disrupted with a significant increase in richness and diversity of microbiota and between-group population differences, while it was partially restored after butyrate supplementation, although the improvement was insignificant but leaning toward the normal control group. The lack of effect on the α-diversity of the gut microbiota after butyrate supplementation found in this study is similar to other interventions documented in the literature. For example, dietary butyrate glycerides and fiber intervention, particularly involving fructans and galactooligosaccharides, had no effect on microbial α-diversity but altered its composition [26, 27]. These findings suggested that single-substance interventions are unlikely to facilitate changes in the α-diversity of the gut microbiota. In addition, to investigate which gut bacteria were regulated by butyrate, we performed LEfSe analysis and showed that the relative abundance of the representative gut microbiota in diabetic mice and the control group was different. A similar phenomenon was also found in a study of the T1D diabetic population and healthy donors [28]. Moreover, some of the representative gut microbiota components of the T1D + NaB group overlapped with those of the control group, indicating that butyrate could partially restore gut microbiota alterations and rebuild a healthy gut microenvironment.

Of eight SCFA concentrations measured in plasma, only butyric acid, caproic acid, and 4-methylvaleric acid significantly decreased in the T1D group compared with the controls, while they increased to some degree after butyrate supplementation. The time from the last treatment to sampling for SCFA analysis is 6 h in our study. Indeed, butyrate plasma clearance is very rapid, when given intravenously its half-life is about 6 min [29]. Sodium butyrate delivered orally to mice at 5 g/kg produced peak plasma butyrate concentrations of approximately 9 mM at 15 min after dosing and plasma butyrate concentrations exceeding 1 mM for 90 min after dosing [30]. In our study, we speculate that the therapeutic effect of sodium butyrate on diabetic retinopathy was the cumulative effect of continuous 12 weeks of administration. In addition, the concentrations of SCFA in the blood we measured were more likely the effect of gut microbiota modification, rather than the result of butyric acid supplementation directly. Of note, butyrate can be used as a substrate to yield a diverse mixture of carboxylic acids, including caproic acid and valeric acid [31, 32]. Under the action of acyl-CoA transferase, hexanoyl-CoA reacted with butyrate to produce caproic acid. When hexanoyl-CoA was sufficient, adding butyric acid and acetic acid produced caproic acid, indicating that hexanoyl-CoA and acetic acid reacted to produce caproic acid [33]. In addition, butyric acid, caproic acid, and valeric acid were identified as histone deacetylase (HDAC) inhibitors [34]. HDAC inhibitors have long been studied as potential therapeutic agents, and there is evidence that microbial-derived HDAC inhibitors are associated with improvements in neurodegenerative and colorectal diseases [35, 36].

Butyrate is produced by the intestinal microbiota and is essential for maintaining host health [13]. A recent study [37] reported that butyrate can cross the blood‒brain barrier and has taken an active part in potential protective therapy [32]. Moreover, butyrate is also intimately related to intestinal inflammation and immunomodulation by increasing the expression of tight junction proteins in colon epithelia and exhibiting anti-inflammatory effects [12]. This is consistent with our findings that the expression of intestinal tight junction proteins (ZO-1 and Occludin) was elevated after butyrate supplementation. Additionally, we found that butyric acid content was decreased in STZ-induced diabetic mice, which is consistent with the results of human population studies [38]. Intriguingly, butyrate supplementation not only increased butyrate levels but also alleviated the degree of DR in STZ-induced diabetic mice, supporting the previously reported protective effects of butyrate on diabetic complications [2]. Alternatively, caproic acid is directly generated by some bacteria, and most caproic acid-producing bacteria are obligate anaerobic bacteria [39]. Caproic acid is a natural antibacterial agent that protects against dysbiosis and the expansion of pathogenic bacteria in animals and enhances animal immunity [39, 40]. In humans, caproic acid is an anti-IL32 and anti-inflammatory player that has recently been shown to be correlated with cardiovascular diseases [41]. 4-methylvaleric acid is known as a precursor of pogostone [42]. Pogostone has anti-inflammatory and immunoregulatory properties and reshapes the gut microbiota. Several researches have demonstrated that pogostone could attenuate TNF-α and lipopolysaccharide-induced cell injury via regulation of the NF-κB and Nrf2 signaling pathways [43, 44]. Additionally, pogostone modulates the gut microbiota and improves the intestinal microenvironment by stimulating SCFA producers, the key SCFA-sensing receptors, and strengthening the epithelial barrier [45]. Therefore, we speculate that butyrate plays the role of regulating the gut barrier and microbiota, protecting the retina by enhancing the interaction of butyric acid, caproic acid, and 4-methylvaleric acid.

Of note, the relative abundances of the *norank_f_Muribaculaceae*, *Ileibacterium*, and *Dubosiella* genera were decreased in the diabetic mice, all of which were positively correlated with the above three SCFAs (butyric acid, 4-methylvaleric acid, and caproic acid) concentrations. Past researchers have reported these three genera to be associated with type 2 diabetes mellitus, obesity, SCFAs, and inflammation in mice [46–48]. Recently, researched have confirmed that the relative abundances of the genera *norank_f_Muribaculaceae* and *Ileibacterium* were negatively correlated with the area under the curve of oral glucose tolerance test in mice [47]. In this work, the elevated blood glucose in mice of the T1D and T1D + NaB groups may be attributed to the lower abundance of *norank_f_Muribaculaceae*, *Ileibacterium*, and *Dubosiella* genera compared with the control group. Additionally, studies have demonstrated that *norank_f_Muribaculaceae* and *Dubosiella* were protective bacterial genera, and both were positively correlated with SCFA levels, especially butyric acid levels, and negatively associated with the mRNA expression of inflammatory factors such as IL-1β, IL-6, and TNF-α [48, 49]. Therefore, it can be speculated that the *norank_f_Muribaculaceae*, *Ileibacterium*, and *Dubosiella* genera are involved in diabetic lesions by influencing the concentration fluctuation of butyric acid, 4-methylvaleric acid, and caproic acid.

Furthermore, only *Lachnospiraceae_NK4A136_group* and *Lactobacillus* genera were significantly negatively correlated with butyric acid, both of them were increased in the T1D group while decreased after butyrate supplementation. The increased *Lachnospiraceae_NK4A136_group* and *Lactobacillus* may contribute to the disturbance of glucose metabolism. In a model of T1D rats, glucose metabolism was downregulated, as characterized by lower levels of pyruvate, citrate, and lactate and higher levels of glucose in serum, but anaerobic glycolysis was enhanced, as characterized by higher levels in the hippocampus [50]. In a model of type 2 diabetic mice, the abundances of *Lachnospiraceae_NK4A136_group* and *Lactobacillus* were positively correlated with the levels of blood glucose and serum glycosylated hemoglobin, whereas negatively correlated with changes in body weight and the quantitative insulin sensitivity check index [51]. It can be speculated that the increased abundance of *Lachnospiraceae_NK4A136_group* and *Lactobacillus* in T1D group is the possible cause of aggravating glucose metabolism disorder, which can be improved by butyrate. Additionally, *Lachnospiraceae_NK4A136_group* is butyrate-producing bacteria. The dominant metabolites of *Lactobacillus* genus lactate can be used as precursors for butyrate synthesis [52]. It is possible that butyrate-producing and lactate-producing bacteria were selectively increased to meet the need for butyric acid in diabetic mice. Due to the extra butyrate supplementation, the demand for butyric acid and related bacteria were decreased.

Our results have potential therapeutic implications. As indicated by our animal studies, supplementation with sodium butyrate not only modulate gut microbiota, but also exerts potent beneficial effects on DR, indicating that supplementation with sodium butyrate is a potential promising therapy for DR. However, this study also had several limitations. First, the sample size of each group of mice was small. Second, in this study, we focused on early diabetes with mild retinopathy rather than late complications. Whether butyrate has the same effects on severe complication of advanced DR remains unclear. Third, fecal samples were collected in the experimental endpoint only, thus we could not observe the dynamic changes in gut microbiota. In order to address these limitations and confirm the findings of this study, a wider sample of animal models, multiple time points, and more detailed molecular biology experiments will be required.

## Conclusion

In conclusion, our study confirmed that sodium butyrate can regulate the composition of the gut microbiota and the disturbance of SCFA metabolites in plasma, increase the expression of intestinal tight junction protein, and thereby effectively delay the progression of DR in T1D mice. Additionally, butyric acid, caproic acid, and 4-methylvaleric acid were the main SCFA that changed in diabetic mice and were regulated by sodium butyrate. Moreover, the above three SCFA and related gut microbiota would support individual prevention and intervention strategies for diabetes with DR. In the end, this study provides a basis for further exploring the mechanism of sodium butyrate to delay DR and provides a new direction for sodium butyrate in the treatment of DR.

## Supplementary Information

**Additional file 1: Figure S1.** Representative electroretinography under different flash intensities is shown for the control (black line), T1D (red line), and T1D + NaB (blue line) groups.

**Additional file 2: Figure S2.** Correlation between the relative abundance of six dominant bacterial genera and 4-methylvaleric acid in plasma. Correlations were calculated in MaAsLin analysis: (A) *Dubosiella* (coefficient = 0.008937, *p* = 0.000508), (B) *Ileibacterium* (coefficient = 0.002501, *p* = 0.000457), (C) *Lactobacillus* (coefficient = − 0.002272, *p* = 2.7E−5), (D) *Escherichia-Shigella* (coefficient =  − 0.005761, *p* = 0.005186), (E) *Bifidobacterium* (coefficient =  − 0.002392, *p* = 0.002686), and (F) *Lachnospiraceae_NK4A136_group* (coefficient =  − 0.005458, *p* = 0.001371). The color and shape of points represent individuals in each group. MaAslin, Multivariate Association with Linear Models.

**Additional file 3: Figure S3.** Correlation between the relative abundance of six dominant bacterial genera and caproic acid in plasma. Correlations were calculated in MaAsLin analysis: (A) *Dubosiella* (coefficient = 0.001693, *p* = 6.072982e−8), (B) *Ileibacterium* (coefficient = 0.00044, *p* = 1.1E−5), (C) *Lactobacillus* (coefficient =  − 0.00028, *p* = 0.004951), (D) *Enterococcus* (coefficient =  − 0.000837, *p* = 0.01208), (E) *Escherichia-Shigella* (coefficient =  − 0.000928, *p* = 0.003662), (F) *Lachnospiraceae_NK4A136_group* (coefficient =  − 0.000752, *p* = 0.007878), and (G) *Lachnospiraceae* (coefficient =  − 0.000172, *p* = 0.007303). The color and shape of points represent individuals in each group. MaAslin, Multivariate Association with Linear Models.

## Data Availability

The data that support the findings of this study can be found at: https://dataview.ncbi.nlm.nih.gov/object/PRJNA938452?reviewer=mv5b9d8l25vfh6g78risqkb9q2, reference number PRJNA938452.
