# Supplementary figures and images for: Sodium butyrate ameliorates diabetic retinopathy in mice via the regulation of gut microbiota and related short-chain fatty acids
Source: J Transl Med. 2023 Jul 7;21:451. doi: 10.1186/s12967-023-04259-4 (PMC10329333; doi:10.1186/s12967-023-04259-4)

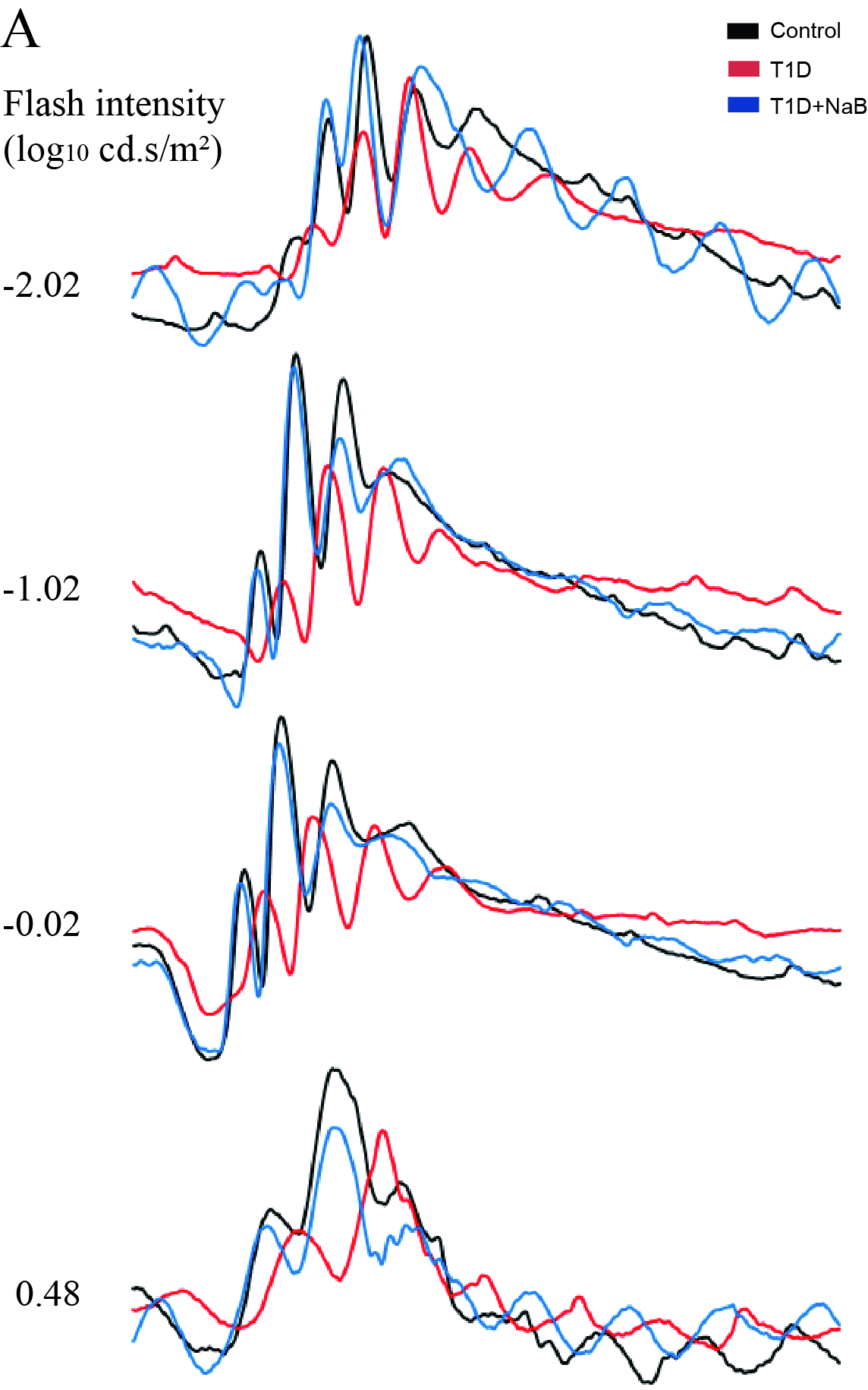

Supplement: Supplementary file 1 — Additional file 1: Figure S1. Representative electroretinography under different flash intensities is shown for the control (black line), T1D (red line), and T1D + NaB (blue line) groups. [file 12967_2023_4259_MOESM1_ESM.tif]

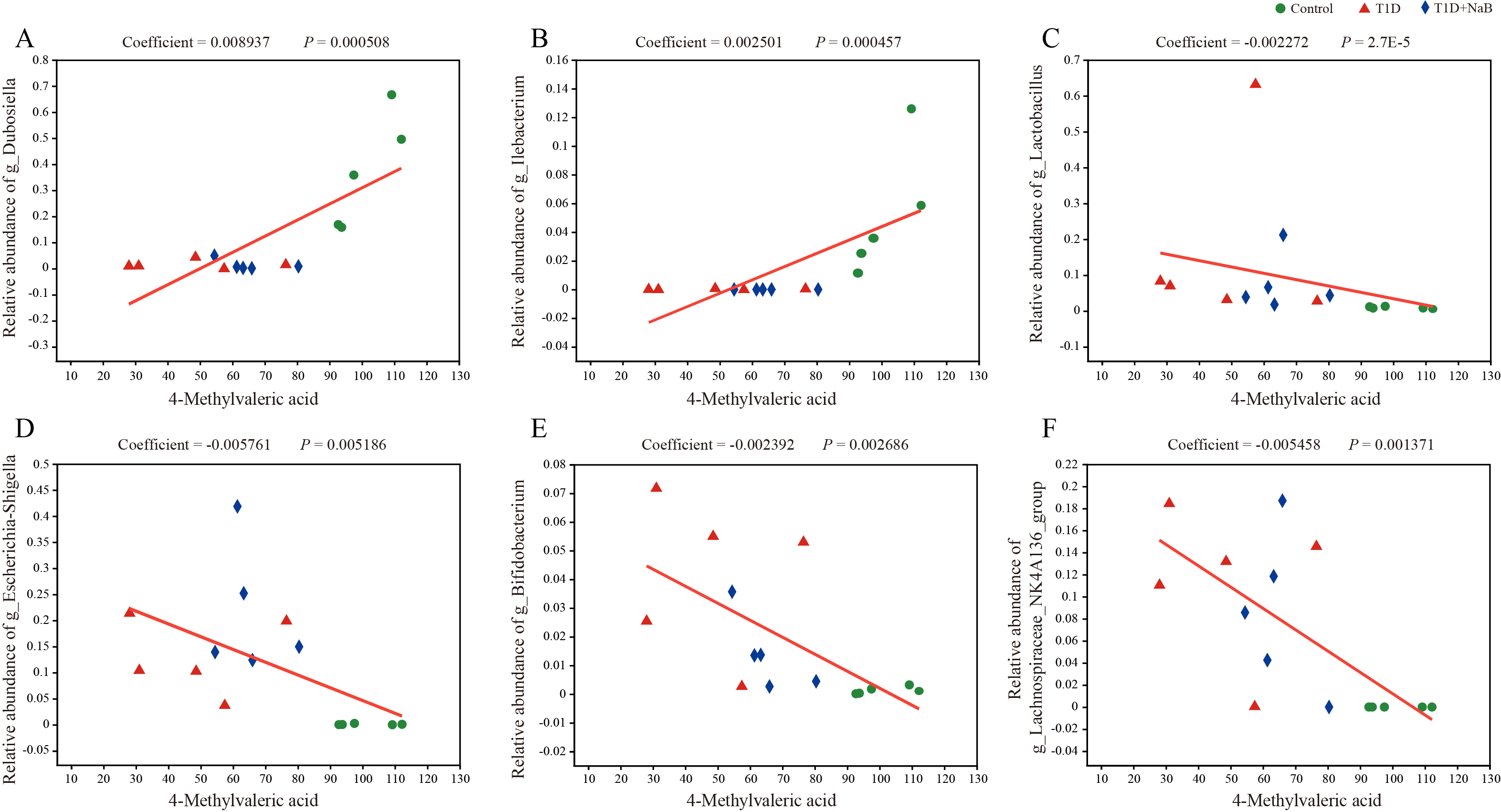

Supplement: Supplementary file 2 — Additional file 2: Figure S2. Correlation between the relative abundance of six dominant bacterial genera and 4-methylvaleric acid in plasma. Correlations were calculated in MaAsLin analysis: (A) Dubosiella (coefficient = 0.008937, p = 0.000508), (B) Ileibacterium (coefficient = 0.002501, p = 0.000457), (C) Lactobacillus (coefficient = − 0.002272, p = 2.7E−5), (D) Escherichia-Shigella (coefficient = − 0.005761, p = 0.005186), (E) Bifidobacterium (coefficient = − 0.002392, p = 0.002686), and (F) Lachnospiraceae_NK4A136_group (coefficient = − 0.005458, p = 0.001371). The color and shape of points represent individuals in each group. MaAslin, Multivariate Association with Linear Models. [file 12967_2023_4259_MOESM2_ESM.tif]

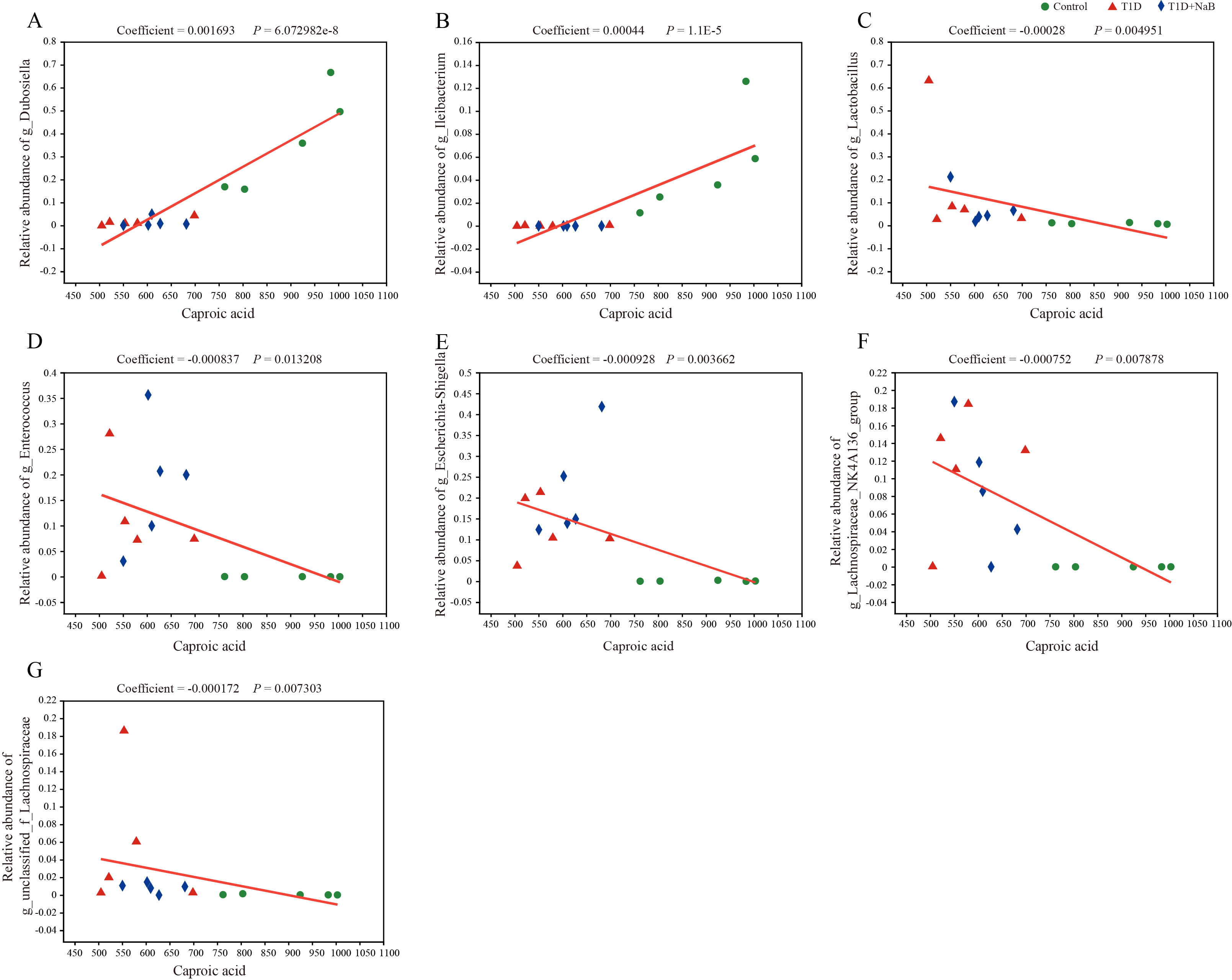

Supplement: Supplementary file 3 — Additional file 3: Figure S3. Correlation between the relative abundance of six dominant bacterial genera and caproic acid in plasma. Correlations were calculated in MaAsLin analysis: (A) Dubosiella (coefficient = 0.001693, p = 6.072982e−8), (B) Ileibacterium (coefficient = 0.00044, p = 1.1E−5), (C) Lactobacillus (coefficient = − 0.00028, p = 0.004951), (D) Enterococcus (coefficient = − 0.000837, p = 0.01208), (E) Escherichia-Shigella (coefficient = − 0.000928, p = 0.003662), (F) Lachnospiraceae_NK4A136_group (coefficient = − 0.000752, p = 0.007878), and (G) Lachnospiraceae (coefficient = − 0.000172, p = 0.007303). The color and shape of points represent individuals in each group. MaAslin, Multivariate Association with Linear Models. [file 12967_2023_4259_MOESM3_ESM.tif]
